# Cancer Incidence and Mortality Among Ethnic German Migrants From the Former Soviet Union

**DOI:** 10.3389/fonc.2018.00378

**Published:** 2018-09-11

**Authors:** Simone Kaucher, Hiltraud Kajüter, Heiko Becher, Volker Winkler

**Affiliations:** ^1^Unit of Epidemiology and Biostatistics, Institute of Public Health, University Hospital Heidelberg, Heidelberg, Germany; ^2^Epidemiological Cancer Registry of North Rhine-Westphalia, Münster, Germany; ^3^Institute for Medical Biometry and Epidemiology, University Medical Center Hamburg-Eppendorf, Hamburg, Germany

**Keywords:** incidence, mortality, migrants, Germany, former soviet union, cohort study, cancer

## Abstract

Germany is a country known for immigration. In 2015, 21% of the general population in Germany consisted of individuals with a migration background. This article focuses on cancer-specific incidence and mortality among one of the biggest migrant groups in Germany: the resettlers. Resettlers are ethnic Germans who mainly immigrated from the Russian federation and other countries of the former Soviet Union after its collapse in 1989. We investigated differences between resettlers and the general German population, regarding (i) incidence and mortality of malignant neoplasms, (ii) time trends of the corresponding incidence and mortality, and (iii) cancer stage at diagnosis. We provide data from two resettler cohorts covering an observation time of 20 years: one cohort on cancer incidence (*N* = 32,972), and another cohort on mortality (*N* = 59,390). Cancer-specific standardized incidence ratios (SIR) and standardized mortality ratios (SMR) for all malignant neoplasms combined and the most common cancer-sites were calculated between resettlers and the general German population. Time trend analyses using Poisson regression were performed to investigate the developments of SIRs and SMRs. To investigate differences in stage at diagnosis, logistic regression was performed, calculating Odds Ratios for condensed cancer stages. We observed higher incidence and mortality of stomach cancer [SIR (men) 1.62, 95%CI 1.17–2.19; SMR (men) 1.62, 95%CI 1.31–2.01; SIR (women) 1.32, 95%CI 0.86–1.94; SMR (women) 1.52, 95%CI 1.19–1.93] and higher mortality of lung cancer [SMR (men) 1.34, 95%CI 1.20–1.50] among resettlers compared to the general German population, but lower incidence and mortality of colorectal (both sexes), lung (women), prostate and female breast cancer. However, time trend analyses showed converging incidence risks of cause-specific incidence over time, whereas differences of mortality did not show changes over time. Results from logistic regression suggest that resettler men were more often diagnosed with advanced cancer stages compared to the Münster population. Our findings suggest that risk factor patterns of the most common cancer-sites among resettlers are similar to those observed within the Russian population. Such increases in prostate, colorectal and breast cancer incidence may be the consequence of improved detection measures, and/or the adaptation of resettlers to the German lifestyle.

## Introduction

In 2015, there were an estimated number of 247.5 million migrants worldwide (1). Research on migrants is important, since it contributes to the knowledge of disease etiology and also reveals differences in the health status between migrants and host populations. Differences in health status are often linked to different exposures in the migrant country of origin (2, 3), to the migration process itself (4) and to integration in the host country (5). Migrants are often a vulnerable group and have a lower socioeconomic status compared to the host population. Consequently, migrants often may have higher risks of diseases that are related to their living and working environment (6). Migrants may seek health care in an altered manner relative to the German population due to their different perceptions of risk, health, and disease combined with poor language skills (7).

Previous research regarding cancer risk among migrants showed heterogeneous results dependent upon cancer-site of interest, country of origin, and host country. In general, it was observed that migrants from non-Western countries showed a higher risk of infectious-related cancer-sites than the host populations of Western European countries, including stomach, liver and cervix uteri cancer. On the contrary, a lower risk of cancer-sites related to a Western lifestyle was observed, including breast and colorectal cancer (8). These results reflect findings from studies in the US and in Australia, which also found a lower breast cancer risk and a higher incidence risk of stomach and liver cancer among migrants compared to host populations (9–11). Furthermore, it was observed that breast cancer risk of non-Western migrants increased with duration of stay and increasing acculturation (9).

Germany has long been a country of immigration (4). In 2015, 21% of the general population in Germany reported to having a migration background. An individual was classified as having a migration background if they or at least one parent immigrated to Germany from their country of origin (12). The two biggest migrant groups in Germany originate from Turkey and the former Soviet Union (FSU). During the early 1960s many Turkish people migrated to Germany for work. However, migrants from the FSU are a unique group of ethnic Germans (resettlers: in German: (Spät-) Aussiedler), whose ancestors emigrated to the Russian empire in the 18th and 19th centuries. After World War II, resettlers were allowed to immigrate to Germany, obtaining German citizenship upon arrival. Consequently, after the collapse of the Soviet Union, many ethnic German migrants immigrated from the FSU (13, 14). To avoid (self-) segregation of incoming resettlers, the German government passed the law of residence assignment in 1989. After arrival, resettlers were usually assigned to their first place of residence based on regional population density and economic performance of the federal state. Although resettlers were obliged to live in this assigned place of residence for at least 2 years (since 2005 3 years) (15), a few exceptions to this rule were permitted. In some circumstances people immigrating to Germany were allowed to resettle closer to family members already living in the country, rather than being assigned to a city by the German government.

Since the late 1980s the FSU has been undergoing massive social changes. These changes have in turn led to a dramatic decrease in life expectancy and overall mortality crisis. In the Russian federation between 1987 and 1994, mortality for all major causes of death (except for cancer) increased (16). This mortality development was very similar to that observed in Kazakhstan and in Ukraine. In 2006, age-adjusted mortality was still high with about 1,300 deaths per 100,000 people compared to 650 per 100,000 people in Germany (17). Table 1 compares age-adjusted estimated incidence and mortality rates of the most common cancer-sites between Germany, the Russian federation and Kazakhstan in 2012 and shows incidence/mortality ratios for each country, indicating survival after cancer diagnosis (18).

**Table 1:** Estimated age-adjusted incidence and mortality rates (adjusted to Segi) per 100,000 and incidence/mortality ratios for all malignant neoplasms and the most common cancer-sites in Germany, the Russian federation and Kazakhstan in 2012 (18).

**Cancer-site**	**Germany (incidence/mortality) ratio**	**Russian federation (incidence/mortality) ratio**	**Kazakhstan (incidence/mortality) ratio**
**MEN**			
All malignant neoplasms^*^	323.7/122.1 2.65	245.8/176.3 1.39	282.2/202.5 1.39
Stomach	10.7/5.7	24.5/20.6	35.2/30.3
	1.88	1.19	1.16
Colorectal	39.7/13.1	30.0/19.9	29.1/16.9
	3.03	1.51	1.72
Lung	38.8/31.3	51.4/47.1	59.2/54.5
	1.24	1.09	1.09
Prostate	77.3/10.4	30.1/12.4	14.9/8.6
	7.43	2.43	1.73
**WOMEN**			
All malignant neoplasms^*^	252.5/83.4 3.03	187.1/91.3 2.05	216.7/104.8 2.07
Stomach	5.4/3.1	10.8/8.7	12.8/10.5
	1.74	1.24	1.22
Colorectal	23.3/8.1	21.8/12.6	19.4/10.7
	2.88	1.73	1.81
Lung	17.9/14.5	6.8/5.6	8.1/7.2
	1.23	1.21	1.13
Breast	91.8/15.5	45.6/17.2	63.0/18.0
	5.92	2.65	3.50

* *Without non-melanoma skin cancer (ICD-10: C44 diagnoses)*.

Given the high burden of lung cancer among males and stomach cancer among both sexes in resettler country of origin, this article focuses on cancer-specific incidence and mortality among resettlers in comparison to the general German population. Furthermore, the development of cancer incidence and mortality will be investigated over 20 years after immigration to Germany and cancer stage at diagnosis will be compared between resettlers and the Münster population.

## Materials and methods

### Cancer incidence

To investigate cancer incidence among resettlers, a registry-based cohort was established in the administrative district of Münster [part of the federal state North Rhine-Westphalia (NRW)], called the AMIN cohort (Aussiedler in Münster - Incidence cohort study). The cohort consists of a sample of all resettlers who were assigned to the study area between 1990 and 2001.

Cancer cases of this cohort were assessed by the federal cancer registry of NRW. The cancer registry performed a pseudonymized record linkage by using encrypted personal identifiers instead of plaintext data (19, 20). Additionally, issues arising from name changes were addressed by utilizing information on common changes from previous studies on ethnic German migrants. Data was collected on the incidence of all cancer cases in the administrative district of Münster between 1994 and 2013, and it was documented whether or not the individual was a cohort member. The study was restricted to histologically confirmed primary malignant tumors (excluding non-melanoma skin cancer). Cancer site-specific analyses were performed for the most common cancer-sites among the resettler cohort (stomach, colorectal, lung, female breast, and prostate cancer).

Since vital status could not be assessed with the follow-up procedure used for the AMOR cohort (described below), person-time was estimated based on an approach for cohorts with an incomplete follow-up (21). In brief, person-time of each individual was first calculated between date of immigration and date of diagnosis or 31st of December 2013 (end of follow-up). In a second step, the person-time estimation procedure used information on out-migration and on non-cancer mortality from the AMOR cohort (described below). Sensitivity analyses were additionally performed to control for possible biases resulting from these assumptions.

### Cancer mortality

Mortality was investigated by combining three registry-based cohort studies on resettlers immigrating between 1990 and 2005, called AMOR studies (Aussiedler Mortality cohort studies). These cohort studies collected data from different regions in Germany: the federal state of NRW (22), the federal state of the Saarland (23) and the region of Augsburg in the federal state of Bavaria (24). Mortality follow-up was performed until 31st of December 2009. Local registry offices provided information on the vital status of each cohort member (alive, deceased). If the status was deceased, cause of death was either retrieved from local health authorities as anonymized death certificates or from regional statistical offices, using ICD codes. A detailed description of the cohorts, the follow-up procedure and the study characteristics, as well as detailed analyses on mortality of the pooled AMOR cohort can be found elsewhere (25).

Person-time was calculated for each individual in one of three ways: either between the date of immigration and the date of death, the date of out-migration or the 31st of December 2009 (end of follow-up). In case of a missing date of event or loss to follow-up, the midpoint between the last known contact and 31st of December 2009 was used as the end of observation. Applying a SAS macro, person-years were calculated to the exact day (26).

To compare resettler cancer mortality to the general German population, the WHO mortality database was used to calculate mortality rates for standardization. Thus, the rates for comparison included observed deaths of the cohort (17).

### Statistical analyses

For all malignant neoplasms combined and the most common cancer-sites, standardized incidence ratios (SIRs) and standardized mortality ratios (SMRs) with exact 95% confidence intervals (95%CI) were calculated. Expected numbers of cancer diagnoses were calculated using incidence rates of the Münster population excluding the resettler cohort and the estimated person-years from the AMIN cohort. Expected numbers of cancer deaths were calculated using mortality rates of the general German population from the WHO mortality database.

Time trends of cancer incidence were analyzed by modeling SIRs with Poisson regression using the observed number as the dependent and year [defined as “calendar year – 1993” (1: 1994, …, 20: 2013)] as the independent variable. The offset was the logarithm of the expected number. Time trends of cancer mortality were modeled accordingly, except defining year as “calendar year – 1989” (1:1990, …, 20:2009).

Cancer stage at diagnosis was categorized in condensed stages as local or advanced based on the T Classification system (T information: tumor size). This system applies cancer-site specific rules of the European Network of cancer registries (ENCR) in order to classify cancer stage (27). For sensitivity analysis the NM Classification system for condensed stage was used (28), which uses information on regional lymph nodes (N) and distant metastasis (M) for each tumor for classification. In the analyses, stomach, colorectal, lung, breast, and prostate cancer were investigated as combined cancer-sites. Since staging classifications differ by cancer-sites and cannot be applied for all cancer-sites (e.g., lymphomas or brain tumors), the analysis was restricted to the most common cancer-sites. Table 2 presents the different classification systems for the observed cancer-sites.

**Table 2:** Condensed classification systems to categorize stages into local, advanced, and unknown stages.

	**T Classification^a^**		**NM Classification^b^**	
**Stage**	**T status *(for stomach, colorectal, lung & prostate cancer)***	**T status *(for breast cancer)***	**N status**	**M status**
Local	T1-T2	T1-T3	N0 N0	M0 Unknown
Advanced	T3-T4	T4	N1-N3 N1-N3 any N	M0 Unknown M1
Unknown	Unknown	Unknown	Unknown Unknown	M0 Unknown

a *Cancer-site specific classification*.

b *Same classification system for the five investigated cancer-sites*.

Logistic regression was used to assess the association between advanced stage at cancer diagnosis and resettler status (yes/no). Condensed stage was the dependent variable and resettler status the independent variable while adjusting for age at diagnosis and year of diagnosis [again defined as “calendar year – 1993” (1: 1994, …, 20: 2013)]. As the main model encompassed a complete case analysis, unknown stages were excluded from the analysis. Additionally, sensitivity models were performed: all unknown stages were assumed to be either (I) local stage or (II) advanced stage.

All statistical analyses were performed separated by sex using SAS Version 9.4.

## Results

### Descriptive results

Table 3 compares the study characteristics of the AMIN and the AMOR cohorts. The AMIN cohort was estimated to accumulate 483,371 person-years with a mean follow-up time of 14.7 years. The AMOR cohort accumulated 797,264 person-years with a mean follow-up time of 13.4 years.

**Table 3:** Study characteristics of the AMIN and the AMOR cohort.

**Characteristics**	**AMIN cohort**		**AMOR cohort**	
	***N***	**%**	***N***	**%**
**NUMBER OF INDIVIDUALS**				
Total	32,972	100.0	59,390	100.0
Men	16,033	48.6	28,744	48.4
Women	16,939	51.4	30,646	51.6
**PERSON-YEARS**				
Total	483,371	100.0	797,264	100.0
Men	234,124	48.4	384,404	48.2
Women	249,247	51.6	412,860	51.8
**IMMIGRATION PERIOD**				
1990-1992	9,363	28.4	17,367	29.2
1993-1995	9,863	29.9	18,637	31.4
1996+	13,746	41.7	23,386	39.4
**AGE AT IMMIGRATION**				
< 18 years	11,598	35.2	9,536	16.1
18–34 years	9,217	28.0	19,604	33.0
35–64 years	10,579	32.1	24,555	41.4
≥65 years	1,578	4.8	5,695	9.6
**Characteristics**	**AMIN cohort**		**AMOR cohort**	
	**Mean (median, range)**		**Mean (median, range)**	
**AGE AT IMMIGRATION**				
Total	29.1 (27.5, 0–99)		36.6 (35, 0-98)	
Men	27.8 (26, 0-92)		35.1 (34, 0-95)	
Women	30.3 (28, 0-99)		38.0 (36, 0-98)	

In both cohorts there were slightly more women than men (~52%) and the distribution of immigrating resettlers by immigration period were comparable. Notably, the AMOR cohort contained relatively more resettlers in the immigration period from 1996 and beyond relative to the AMIN cohort. It was also revealed that the study population of the AMIN cohort was younger compared to the mortality cohort.

Between 1994 and 2013, 3.9% (*N* = 1,291) of the AMIN cohort individuals were diagnosed with a primary malignant tumor of which 87.6% (*N* = 1,131) were histologically confirmed. The five most frequent cancer diagnoses were breast cancer (*N* = 183, 16.2%), colorectal cancer (*N* = 155, 13.7%), lung cancer (*N* = 107, 9.5%), prostate cancer (*N* = 106, 9.4%), and stomach cancer (*N* = 69, 6.1%).

Follow-up was complete for 95.2% of the AMOR cohort and information regarding the cause of death was available for 92.2% of the 5,572 observed deaths. Altogether, 1,533 deaths due to malignant neoplasms were observed, whereof the three most common cancer-sites were lung (*N* = 369), colorectal (*N* = 169), and stomach (*N* = 150).

### SIR and SMR analyses

Table 4 shows results of the SIR and SMR analyses for men and women as well as age-standardized mortality rates for the general German population. Cancer incidence for all malignant neoplasms combined was lower among resettlers compared to the Münster population, for both sexes respectively. While cancer mortality for all combined malignant neoplasms was lower among resettler women compared to the general German population, no differences were observed among men.

**Table 4:** Standardized incidence ratios (AMIN cohort, 1994-2013) and standardized mortality ratios (AMOR cohort, 1990-2009) with exact 95% confidence intervals and age-standardized mortality rates for Germany (1990-2009) for all malignant neoplasms combined and the most common cancer-sites, separated by sex.

		**AMIN cohort (1994-2013)**		**AMOR cohort (1990-2009)**		**Germany (1990-2009)**
**Cause**	**ICD-10 code**	**Observed diagnoses**	**SIR (95%CI)**	**Observed deaths**	**SMR (95%CI)**	**Mortality rates^a^**
**MEN**						
Malignant neoplasms…^*^	C00-C97	556	**0.87** **(0.80–0.95)**	864	1.00 (0.94–1.07)	237.2
… of stomach	C16	43	**1.62** **(1.17–2.19)**	84	**1.62** **(1.31–2.01)**	15.3
… of colorectal organs	C18-C21	78	0.82 (0.66–1.03)	77	**0.74** **(0.59–0.93)**	29.2
… of lung, bronchus and trachea	C33-C34	94	1.02 (0.83–1.24)	307	**1.34** **(1.20–1.50)**	61.2
… of prostate	C61	106	**0.72** **(0.60–0.88)**	46	**0.58** **(0.42–0.77)**	24.8
**WOMEN**						
Malignant neoplasms…^*^	C00-C97	575	**0.82** **(0.75–0.89)**	669	**0.84** **(0.78–0.91)**	145.4
… of stomach	C16	26	1.32 (0.86–1.94)	66	**1.52** **(1.19–1.93)**	8.3
… of colorectal organs	C18-C21	77	**0.79** **(0.64–0.99)**	92	0.86 (0.70–1.05)	19.5
… of lung, bronchus and trachea	C33-C34	13	**0.30** **(0.16–0.51)**	62	**0.69** **(0.54–0.88)**	15.4
… of breast	C50	183	**0.70** **(0.60–0.81)**	82	**0.55** **(0.44–0.68)**	28.4

* *Without non-melanoma skin cancer (ICD-10: C44 diagnoses)*.

a *Per 100,000 inhabitants, using European standard population (29)*.

*Significant results are bolded*.

Stomach cancer incidence and mortality was found to be higher among resettlers compared to the general population, for both sexes respectively. In contrast, resettlers showed lower incidence and mortality of prostate and female breast cancer than that observed within the general populations. Among resettler men, a significantly lower mortality of colorectal cancer was observed, whereas resettler women showed a significant lower incidence of colorectal cancer. There was no difference observed regarding lung cancer incidence among men, however, lung cancer mortality was higher compared to the general population. Resettler women showed both lower lung cancer incidence and mortality than the general populations.

### Time trend analyses

**Figure 1:**
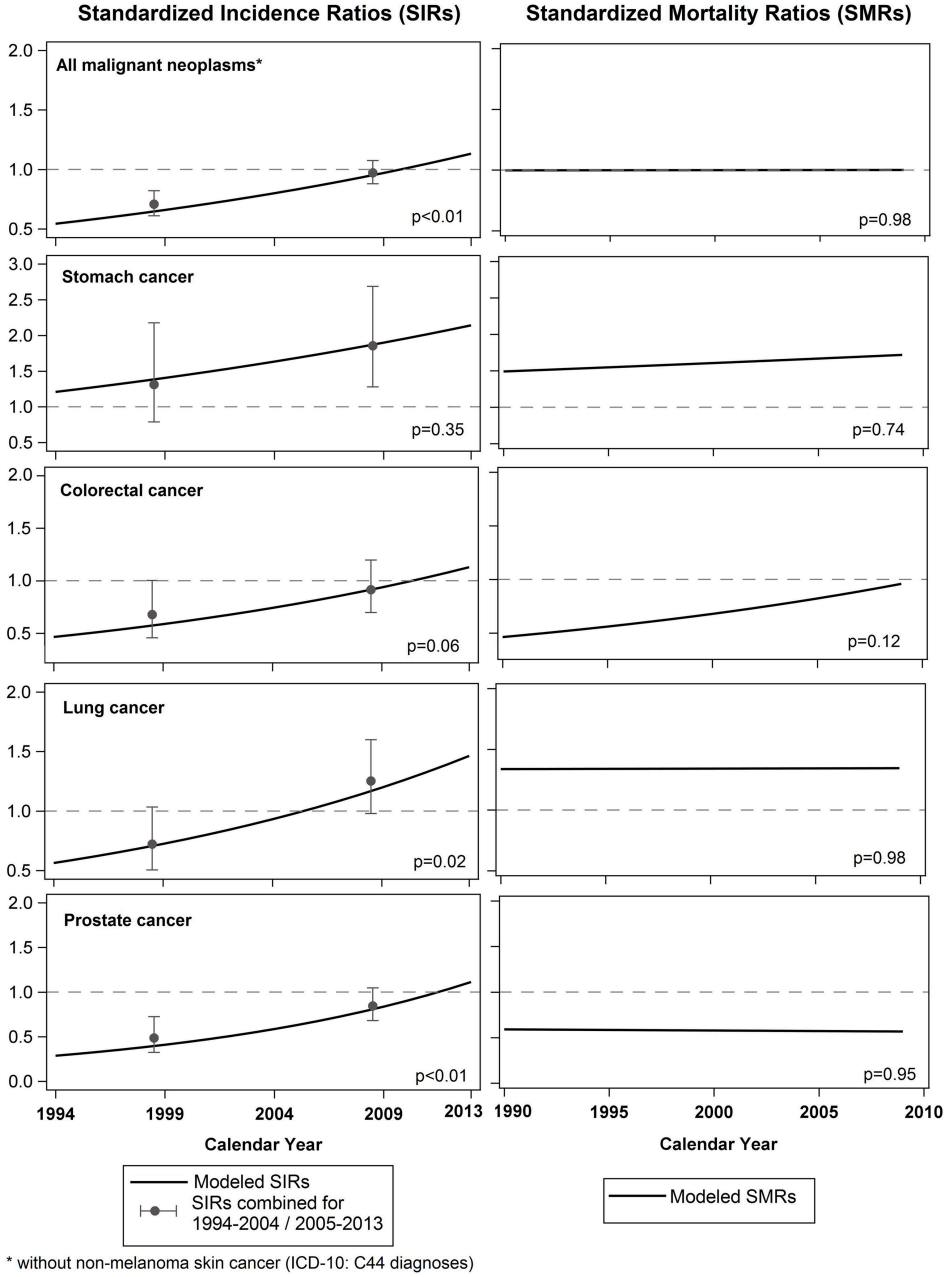
Standardized incidence ratios (AMIN Cohort, 1994-2013) & standardized mortality ratios (AMOR Cohort, 1990-2009) for all malignant neoplasms combined and the most common cancer-sites for men with corresponding *p*-values of the linear calendar year effect of the Poisson model.

Figure 1 shows modeled SIRs from 1994 to 2013 and modeled SMRs from 1990 to 2009 for men. SIRs combined for two time-periods (1994-2004 and 2005-2013) have been added to the figure, as well as *p*-values of the linear calendar year effect of the Poisson model.

Whereas the incidence risk of all malignant neoplasms combined was lower among resettlers and converged to the incidence risk of the Münster population until the end of observation period, the mortality risk of all malignant neoplasms combined remained unchanged between resettlers and the general German population during the observation period.

Stomach cancer incidence and mortality did not reveal any significant effect over time, whereas lung, colorectal and prostate cancer incidence risks were found to be lower among resettler men than in the German population. Until 2013, the incidence risk of colorectal and prostate cancer converged to the incidence risk of the Münster population. Differences in colorectal and prostate cancer mortality did not show any time effects. The incidence risk of lung cancer converged to the incidence risk of the Münster population up until 2005. Afterwards, lung cancer incidence risk among resettler men further increased. For lung cancer mortality, a remaining higher mortality was observed among resettlers compared to Germans.

**Figure 2:**
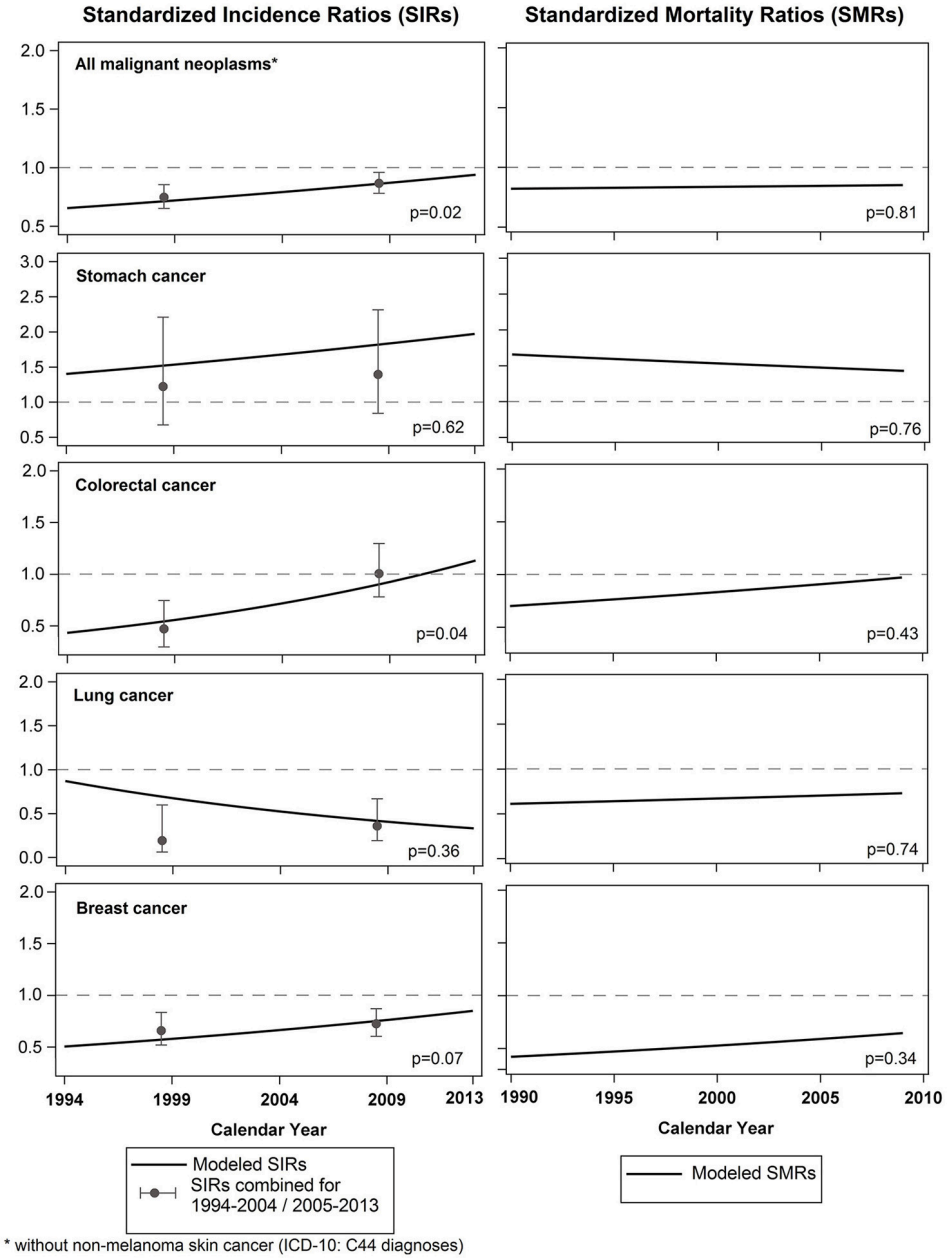
Standardized incidence ratios (AMIN Cohort, 1994-2013) & standardized mortality ratios (AMOR Cohort, 1990-2009) for all malignant neoplasms combined and the most common cancer-sites for women with corresponding *p*-values of the linear calendar year effect of the Poisson model.

Figure 2 shows modeled SIRs from 1994 to 2013 and modeled SMRs from 1990 to 2009 for women. Again, SIRs combined for two time periods (1994-2004 and 2005-2013) have been added to the figure, as well as *p*-values of the linear calendar year effect of the Poisson model.

Among women, the incidence risk of all malignant neoplasms combined also converged to the incidence risk of the Münster population, while differences in mortality of all malignant neoplasms combined did not show any time effects. The mortality risk among resettler women remained lower than in the general German population.

A significant change of cancer incidence risk over time was only found for colorectal cancer. Whereas colorectal cancer incidence was lower among resettler women compared to German women in 1994, the incidence risk converged to that of the Germans until 2013. Breast cancer incidence risk was found to be increasing among resettler women over time, however, the effect was not significant. Stomach and lung cancer incidence risk among resettler women did not show an effect over time. Cancer-site specific mortality time trends did not show any significant effect during the observation time.

### Cancer stage analyses

Table 5 presents the distribution of local, advanced and unknown stages for combined cancer-sites, separated by resettlers and the Münster population (without resettler cohort) and for men and women. The distribution of cancer stage of the most common cancer-sites can be found in the Supplementary Table 1. The tables compare cancer stage according to two different applied classification systems. In general, the distribution of stages by the two applied classification systems did not reveal major differences, with the exception of the stage of female breast cancer. The T Classification system showed in general a slightly higher percentage of local cancer stages, whereas the NM Classification system showed a slightly higher percentage of unknown stages. However, no significant difference regarding the stages between resettlers and the Münster population was found in both classification systems, except for women when the T Classification system was applied.

**Table 5:** Distribution of local, advanced and unknown stages of the most common cancer-sites combined (AMIN cohort, 1994-2013), separated by the two classification systems and sex.

	**T classification**				**NM classification**			
**Cancer stage**	**Resettlers**		**Münster population**		**Resettlers**		**Münster population**	
	***N***	**%**	***N***	**%**	***N***	**%**	***N***	**%**
**MEN**								
Local	106	33.0	21,688	33.8	91	28.4	20,301	31.7
Advanced	112	34.9	22,175	34.6	107	33.3	18,146	28.3
Unknown	103	32.1	20,240	31.6	123	38.3	25,656	40.0
**WOMEN**								
Local	198	66.2	34,152	56.9	131	43.8	24,651	41.1
Advanced	60	20.1	14,535	24.2	118	39.5	22,490	37.5
Unknown	41	13.7	11,370	18.9	50	16.7	12,916	21.5

Due to the limited number of observations per specific cancer-site group, adjusted ORs from logistic regression were solely analyzed for combined cancer-sites and are shown in Table 6. In the complete case analysis and the sensitivity analysis II (unknown stage = advanced stage) it was observed that resettler men had higher odds of being diagnosed with an advanced stage than the Münster population, for both classification systems respectively. Among males, the sensitivity analysis I (unknown stage = local stage) showed no differences when the T Classification was applied, while an elevated OR was revealed with the NM Classification system. However, the effect was only significant within the complete case analysis and sensitivity analysis II when the NM Classification was applied. In general, it was observed that the NM Classification showed stronger effects than the T Classification.

**Table 6:** Odds ratios for resettlers being diagnosed with an advanced tumor (AMON cohort, 1994–2013), separated by the two classification systems and sex.

	**T classification**		**NM classification**	
**Model**	**OR^1^ (95%CI)**	***p*-value**	**OR^1^ (95%CI)**	***p*-value**
**MEN**				
Complete case analysis (unknown stages excluded)	1.11 (0.85–1.44)	0.47	**1.45** **(1.10–1.93)**	**0.01**
Sensitivity I (unknown stage = local stage)	0.96 (0.76–1.21)	0.74	1.20 (0.95–1.52)	0.13
Sensitivity II (unknown stage = advanced stage)	1.21 (0.95–1.53)	0.12	**1.42** **(1.11–1.81)**	**0.01**
**WOMEN**				
Complete case analysis (unknown stages excluded)	0.83 (0.62–1.12)	0.22	1.05 (0.82–1.34)	0.72
Sensitivity I (unknown stage = local stage)	0.86 (0.65–1.16)	0.32	1.08 (0.86–1.37)	0.50
Sensitivity II (unknown stage = advanced stage)	**0.78** **(0.61–0.99)**	**0.04**	0.99 (0.79–1.25)	0.92

1 *Adjusted for age at diagnosis and calendar year*.

*Significant results are bolded*.

For women, results showed no difference regarding the cancer stage at diagnosis when the NM classification was applied. Results for women from the T classification suggest that women showed lower odds of being diagnosed with an advanced stage compared to the Münster population. However, the effect was only significant within the sensitivity analysis II.

## Discussion

### Key findings

We found lower incidence of all malignant neoplasms combined among resettlers (both sexes) compared to the Münster population. While mortality of all malignant neoplasms combined was lower among resettler women as well, no difference was observed among men. However, cancer-site specific analyses showed different results: we observed higher stomach cancer incidence and mortality among both male and female resettlers compared to the general population. Furthermore, lung cancer mortality was observed to be higher among resettler men than among men of the general German population. While stomach cancer incidence (both sexes) did not develop differently compared to the Münster population, lung cancer incidence (men) showed increasing disparity over time. Colorectal, lung (female), prostate and female breast cancer incidence was initially found to be lower among resettlers, but the incidence of these cancers converged to the risk of the Münster population over time, with the exception of female lung cancer which remained stable. Mortality time trends showed no significant changes over time for both sexes. Results from logistic regression suggest that resettler men were more often diagnosed with advanced cancer stages compared to the Münster population.

### Shortcomings and limitations

As the cohorts consist of secondary data, information pertaining to common risk factors such as lifestyle and behavior, health care seeking behavior, infections, education, occupation, and parity were unavailable. Further, the incomplete follow-up of the AMIN cohort and consequently, the estimated person-years of the cohort have to be mentioned. However, the applied estimation procedure was found to be valid and reliable (21). Even though sensitivity analyses on out-migration showed only minor differences in SIRs (data not shown), some uncertainty remains regarding the assumptions on out-migration and mortality.

The incidence follow-up may not have identified all resettler diagnoses due to the possibility of name changes among resettlers. To help correct for this, common Russian-German name translations were considered (as reported in previous cohort studies) (30). Diagnoses among resettlers were more likely to be histologically confirmed than diagnoses among the Münster population, an observation which was particularly pronounced within the early observation period. This discrepancy in diagnoses may be due to reporting differences within the two populations. These differences diminished with time, and in 2005 mandatory reporting was introduced which led to a further increase in histological confirmation (31). Even though results of all combined malignant diagnoses and histologically confirmed diagnoses were similar, all analyses were restricted to histologically confirmed diagnoses to minimize the possibility of bias.

Another limitation which should be mentioned is the fact that the reported incidence and mortality analyses are based on different cohorts utilizing different standard populations. The standard populations resemble the respective populations, for cancer incidence the population of the administrative district of Münster in the federal state of NRW and for cancer mortality the general German population. A comparison between the standard populations shows slightly higher cancer incidence as well as slightly higher cancer mortality rates in NRW (32). Further, the incidence comparison was done to the Münster population excluding the study population, while mortality was compared to the general German population, which includes the study population. However, it was shown that effects on the SMR are very small (30). Both cohorts result in a 20 year observation period and overlap for 15 years. Although the cohort studies were conducted in different regions, the introduced bias is expected to be neglectable. After arrival, resettlers were quasi-randomly assigned to their first place of residence based on regional population density and economic performance of the federal states (14). Therefore, the resettler cohorts reflect all resettlers from the FSU living in Germany.

### Integration into the current understanding of the problem

In many migrant studies the healthy migrant effect can at least partly explain a lower mortality among migrants compared to the host population. However, it needs to be emphasized that this is an unlikely explanation for our findings. Resettlers are considered to be a special kind of migrant. Resettlers possessed an invitation to return to Germany irrespective of their qualifications or health status. Upon arrival in Germany, resettlers received German citizenship, access to health care and social system benefits (14). In our study it was observed that resettlers typically did not move to Germany alone; many immigrated to Germany bringing relatives with them. It is assumed that most ethnic Germans moved to Germany (33). Thus, we do not think that a selection of healthy resettlers during the migration process occurred. It is however possible that the “fittest” migrants migrated shortly after 1989.

#### Incidence and mortality

The higher incidence and mortality of stomach cancer among resettlers might be associated with a higher prevalence of a previous helicobacter pylori (h. pylori) infection or with an unhealthy diet (low intake of fruits and vegetables, higher intake of salty, and smoked food) (34). The prevalence of h. pylori infection was found to be higher in individuals belonging to countries of the FSU compared to those in Germany (35). However, a previous study on stomach cancer incidence among resettlers found that higher stomach cancer incidence cannot be explained solely by previous h. pylori infection (36). In addition to dietary composition and obesity, smoking behavior, alcohol consumption and lack of physical activity were found to increase the risk of stomach cancer (34). A previous case-control study on risk factors among resettlers found lower alcohol consumption among resettlers compared to the native German population and no differences regarding fruit and vegetable consumption between resettlers and the German population. Overweight and physical inactivity were found to be more prevalent among resettler women than in German women (37).

Differences in lung cancer mortality may be due to high tobacco smoking prevalence among male and low prevalence among female resettlers. Worldwide, tobacco smoking prevalence in countries of the FSU are among the highest for men but low for women (38). Furthermore, a higher smoking prevalence was found among resettlers compared to the German population (39).

Lower female breast cancer incidence and mortality might mainly be explained by lower age at first pregnancy, higher parity, and lower smoking prevalence as seen in women from FSU countries compared to German women (40). A possible lower participation in the Mammography Screening Program might explain lower incidence, but not lower mortality.

Prevalence of specific lifestyle factors among resettlers may have changed over time. For example, it was observed that smoking behavior decreased among resettler men and increased among women with duration of stay and converged to the smoking rates of the German population (39). This might partly explain the increasing incidence risk of female breast cancer among resettlers, but lung cancer among female resettlers does not yet increase. Additionally, it was observed that the fertility rate among resettlers dropped after arrival in Germany and was found to be even lower than that of German women (41). This might also explain the converging breast cancer incidence. Increasing time trends for colorectal and prostate cancer among men and breast cancer among women further indicate a change of obesity prevalence and dietary composition, which was found previously among resettler women (37). Back in the countries of the FSU, resettlers suffered from food shortages and later on, availability of food was restricted (42). It might be possible that resettlers changed their dietary habits completely once in Germany, due to greater availability and selection of food.

#### Cancer stage at diagnosis

Analysis of cancer stage at diagnosis did result in higher odds of advanced stages among resettler men, corroborated by a sensitivity analysis using more of the available data. Similar results were seen in another study among resettlers (28). The two classification systems are structurally different. The NM classification system easily defines local stages with a small tumor size as unknown stage, since small tumors are more prone for missing information on N and M. In contrast, the T classification tends to define advanced as local stages, since it ignores the fact that even small tumors might have spread. Therefore, results from both classification systems were reported, representing a sensitivity analysis with two slightly biased results. Cancer-site specific results did not show significant effects (data not shown), probably due to the small numbers of events overall. Nevertheless, resettler men seem to have a higher chance of getting a cancer diagnosis at an advanced stage than men of the Münster population. Tumor diagnoses at an advanced stage indicate delay in diagnosis, which might be explained by lower uptake of early detection and screening measures. The greater availability of screening measures during the years might explain the significant decreasing odds of having an advanced stage at diagnoses with increasing calendar year. Since our analyses are based on registry data, we do not know whether the possible lower uptake of early detection and screening measures is due to barriers in access to health care, the lack of knowledge of health care services or due to different health beliefs of resettlers. Spallek et al. reported that participation in prevention programs is lower among specific migrant groups in Germany, however, reasons for that need to be investigated in the future (7).

### Future direction of the research

Following the results of this study, it is important to investigate risk factor patterns among resettlers, including dietary habits, *H. pylori* infection, physical activity, alcohol consumption, and smoking behavior. In addition, information on education and occupation as well as (epi-) genetic factors should be assessed. The NAKO may become useful for this: it is a large prospective cohort study in Germany, which currently recruits 200,000 representative participants. This study will assess lifestyle and environmental factors, and will investigate (epi-) genetic factors (43). Preliminary data from selected study centers indicate that the NAKO study includes about 2% resettlers which will allow more detailed analyses in that direction.

Additionally, a resettler-specific survey study should be conducted, to investigate key lifestyle, environmental and socioeconomic factors among resettlers. Methods leading to better knowledge regarding early cancer detection practices, access to health care and overall necessity thereof may improve the incidence of early cancer detection in the resettler population and hence should be further investigated.

## Data availability statement

Data of the AMIN and AMOR studies are not open access, but we encourage other researchers to contact us and apply for data access based on collaborating projects.

## Author contributions

The cohort studies were initiated by HB and VW and performed by HB, VW, and SK. SK did the statistical analyses and drafted the manuscript. HK provided the incidence data of the AMIN cohort. SK, HK, HB, and VW contributed to writing and editing the manuscript and to the interpretation of the results.

### Conflict of interest statement

The authors declare that the research was conducted in the absence of any commercial or financial relationships that could be construed as a potential conflict of interest.
