# Supplementary material for: Cancer Incidence and Mortality Among Ethnic German Migrants From the Former Soviet Union
Source: Front Oncol. 2018 Sep 11;8:378. doi: 10.3389/fonc.2018.00378 (PMC6141734; doi:10.3389/fonc.2018.00378)
Supplement: Supplementary file 1 [file Table_1.DOCX]

Supplementary table

Distribution of local, advanced and unknown stages of the most common cancer-sites (AMIN Cohort, 1994-2013), separated by the two classification systems and sex

|  |  |  | T Classification | | | | NM Classification | | | |
| --- | --- | --- | --- | --- | --- | --- | --- | --- | --- | --- |
| Sex | Cancer-site | Cancer stage | Resettlers | | Münster population | | Resettlers | | Münster population | |
|  |  |  | N | % | N | % | N | % | N | % |
| Men | Stomach cancer | Local | 12 | 27.9% | 1,399 | 30.8% | 7 | 16.3% | 836 | 18.4% |
|  |  | Advanced | 16 | 37.2% | 1,501 | 33.1% | 22 | 51.2% | 2,028 | 44.7% |
|  |  | Unknown | 15 | 34.9% | 1,640 | 36.1% | 14 | 32.6% | 1,676 | 36.9% |
|  | Colorectal cancer | Local | 21 | 26.9% | 3,636 | 22.2% | 33 | 42.3% | 6,303 | 38.4% |
|  |  | Advanced | 46 | 59.0% | 9,853 | 60.1% | 35 | 44.9% | 6,652 | 40.5% |
|  |  | Unknown | 11 | 14.1% | 2,920 | 17.8% | 10 | 12.8% | 3,454 | 21.1% |
|  | Lung cancer | Local | 26 | 27.7% | 4,460 | 27.8% | 12 | 12.8% | 2,113 | 13.2% |
|  |  | Advanced | 31 | 33.0% | 5,136 | 32.0% | 44 | 46.8% | 7,553 | 47.1% |
|  |  | Unknown | 37 | 39.4% | 6,452 | 40.2% | 38 | 40.4% | 6,382 | 39.8% |
|  | Prostate cancer | Local | 47 | 44.3% | 12,027 | 44.8% | 39 | 36.8% | 10,949 | 40.8% |
|  |  | Advanced | 19 | 17.9% | 5,629 | 21.0% | 6 | 5.7% | 1,817 | 6.8% |
|  |  | Unknown | 40 | 37.7% | 9,194 | 34.2% | 61 | 57.6% | 14,084 | 52.5% |
| Women | Stomach cancer | Local | 8 | 30.8% | 915 | 28.5% | 6 | 23.1% | 550 | 17.1% |
|  |  | Advanced | 10 | 38.5% | 874 | 27.2% | 11 | 42.3% | 1,201 | 37.4% |
|  |  | Unknown | 8 | 30.8% | 1,425 | 44.4% | 9 | 34.6% | 1,463 | 45.5% |
|  | Colorectal cancer | Local | 25 | 32.5% | 3,215 | 20.6% | 35 | 45.5% | 5,844 | 37.4% |
|  |  | Advanced | 38 | 49.4% | 9,185 | 58.8% | 24 | 31.2% | 6,058 | 38.8% |
|  |  | Unknown | 14 | 18.2% | 3,214 | 20.6% | 18 | 23.4% | 3,712 | 23.8% |
|  | Lung cancer | Local | 5 | 38.5% | 1,658 | 27.3% | 0 | 0.0% | 753 | 12.4% |
|  |  | Advanced | 3 | 23.1% | 1,872 | 30.8% | 8 | 61.5% | 2,880 | 47.4% |
|  |  | Unknown | 5 | 38.5% | 2,548 | 41.9% | 5 | 38.5% | 2,445 | 40.2% |
|  | Breast cancer | Local | 160 | 87.4% | 28,364 | 80.7% | 90 | 49.2% | 17,504 | 49.8% |
|  |  | Advanced | 9 | 4.9% | 2,604 | 7.4% | 75 | 41.0% | 12,351 | 35.1% |
|  |  | Unknown | 14 | 7.7% | 4,183 | 11.9% | 18 | 9.8% | 5,296 | 15.1% |
